# Energetics of the coupled electronic–structural transition in the rare-earth nickelates

**DOI:** 10.1038/s41535-019-0145-4

**Published:** 2019-02-06

**Authors:** Alexander Hampel, Peitao Liu, Cesare Franchini, Claude Ederer

**Affiliations:** 1 Materials Theory, ETH Zürich, Wolfgang-Pauli-Strasse 27, 8093 Zürich, Switzerland; 2 Faculty of Physics, Computational Materials Physics, University of Vienna, Vienna, A-1090 Austria; 3 Dipartimento di Fisica e Astronomia, Università di Bologna, 40127 Bologna, Italy

**Keywords:** Electronic properties and materials, Theory and computation

## Abstract

Rare-earth nickelates exhibit a metal–insulator transition accompanied by a structural distortion that breaks the symmetry between formerly equivalent Ni sites. The quantitative theoretical description of this coupled electronic–structural instability is extremely challenging. Here, we address this issue by simultaneously taking into account both structural and electronic degrees of freedom using a charge self-consistent combination of density functional theory and dynamical mean-field theory, together with screened interaction parameters obtained from the constrained random phase approximation. Our total energy calculations show that the coupling to an electronic instability toward a charge disproportionated insulating state is crucial to stabilize the structural distortion, leading to a clear first order character of the coupled transition. The decreasing octahedral rotations across the series suppress this electronic instability and simultaneously increase the screening of the effective Coulomb interaction, thus weakening the correlation effects responsible for the metal–insulator transition. Our approach allows to obtain accurate values for the structural distortion and thus facilitates a comprehensive understanding, both qualitatively and quantitatively, of the complex interplay between structural properties and electronic correlation effects across the nickelate series.

## Introduction

Complex transition metal oxides exhibit a variety of phenomena, such as, e.g., multiferroicity,^1^ non-Fermi liquid behavior,^2^ high-temperature superconductivity,^3^ or metal–insulator transitions (MIT),^4^ which are not only very intriguing, but are also of high interest for future technological applications.^5–7^ However, the quantitative predictive description of these materials and their properties represents a major challenge for modern computational materials science, due to the importance of electronic correlation effects as well as due to the intimate coupling between electronic, magnetic, and structural degrees of freedom.^4,8^

An example, which has received considerable attention recently, is the family of rare-earth nickelates, *R*NiO_3_, with *R* = La–Lu and Y, which exhibit a rich phase diagram that is highly tunable by strain, doping, and electromagnetic fields.^9–14^ All members of the nickelate series (except LaNiO_3_) exhibit a MIT as a function of temperature, which is accompanied by a structural distortion that lowers the space group symmetry from orthorhombic *Pbnm*, where all Ni sites are symmetry-equivalent, to monoclinic *P*2_1_/*n*, with two inequivalent types of Ni sites.^15–18^ The structural distortion results in a three-dimensional checkerboard-like arrangement of long bond (LB) and short bond (SB) oxygen octahedra surrounding the two inequivalent Ni sites (see Fig. 2a), and corresponds to a zone-boundary breathing mode of the octahedral network with symmetry label $R_1^ +$.^19^ In addition, all systems exhibit antiferromagnetic (AFM) order at low temperatures.^9,20,21^ For *R* from Lu to Sm, the AFM transition occurs at lower temperatures than the MIT, whereas for *R* = Nd and Pr, the magnetic transition coincides with the MIT. AFM order in LaNiO_3_ was only reported recently^21^ and is still under discussion.^22^ Due to challenges in synthesis, experimental data on the bulk materials is relatively sparse, and quantitative predictive calculations are therefore highly valuable to gain a better understanding of the underlying mechanisms.

Different theoretical and computational approaches have highlighted different aspects of the coupled structural–electronic transition in the nickelates, thereby focusing either on structural or electronic aspects.^23–30^ Density functional theory plus Hubbard *U* (DFT + *U*) calculations have recently emphasized the coupling between the breathing mode and other structural distortions such as octahedral rotations, as well as the effect of magnetic order.^28–30^ However, these calculations cannot properly describe the transition from the paramagnetic metal to the paramagnetic insulator observed in all nickelates with *R* cations smaller than Nd, and thus cannot correctly capture the important electronic instability. Using DFT plus dynamical mean-field theory (DFT + DMFT),^31^ the MIT has been classified as site-selective Mott transition,^23^ where an electronic instability drives the system toward a charge- (or bond-) disproportionated insulator.^26^ However, the capability of DFT + DMFT to address structural properties is currently not well established, even though promising results have been achieved in previous work,^24,25,27^ employing either simplified interpolation procedures between different structures, fixing lattice parameters to experimental data, or using ad hoc values for the interaction parameters.

Here, we combine a systematic analysis of the structural energetics, with an accurate DFT + DMFT-based description of the electronic structure, using screened interaction parameters obtained within the constrained random phase approximation (cRPA).^32^ Our analysis thus incorporates both structural and electronic effects, and leads to a transparent and physically sound picture of the MIT in the nickelates, which also allows to obtain accurate structural parameters across the whole series. We find that the electronic instability is crucial to stabilize the breathing mode distortion by essentially “renormalizing” the corresponding total energy surface, resulting in a coupled structural–electronic first order transition. Trends across the series are driven by the degree of octahedral rotations,^28^ which control both the strength of the electronic instability as well as the magnitude of the screened interaction parameters.

## Results

### Relaxation of *Pbnm* structures and definition of correlated subspace

All systems are fully relaxed within the high-temperature *Pbnm* space group using nonspinpolarized DFT calculations. We then use symmetry-based mode decomposition^33^ to analyze the relaxed *Pbnm* structures and quantify the amplitudes of the various distortion modes. The mode decomposition allows for a clear conceptional distinction between different structural degrees of freedom, which enables us to obtain those structural degrees of freedom for which correlation effects are not crucial from standard DFT calculations, while the important breathing mode distortion is then obtained from DFT + DMFT total energy calculations. For further details on the DFT results and our distortion mode analysis we refer to our previous work.^30^

Next, we construct a suitable low-energy electronic subspace, for which the electron–electron interaction is treated within DMFT. Here, we follow the ideas of ref. ^26^, and construct Wannier functions only for a minimal set of bands with predominant Ni-*e*_*g*_ character around the Fermi level, which in all cases (except LaNiO_3_) is well separated from other bands at lower and higher energies. The Wannier functions are then used as localized basis orbitals to construct the effective impurity problems for our fully charge self-consistent (CSC) DFT + DMFT calculations,^34^ where the LB and SB Ni sites are treated as two separate impurity problems (even for zero $R_1^ +$ amplitude) coupled through the DFT + DMFT self-consistency loop, and the system is constrained to remain paramagnetic. More details on the construction of the Wannier functions and the technical aspects of our CSC DFT + DMFT calculations can be found in the “Methods” section.

### (*U*, *J*) Phase diagrams

**Fig. 1:**
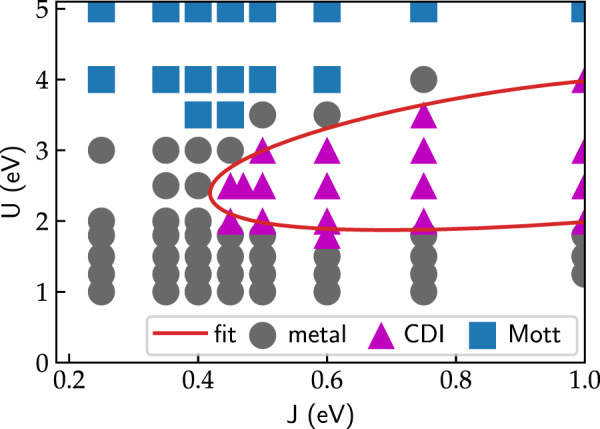
Phase diagram as a function of interaction parameters *U* and *J* for the relaxed *Pbnm* structure of LuNiO_3_, i.e., $R_1^ + = 0.0\,{\text{{\AA}}}$. Each calculation is represented by a marker. Three different phases can be identified, indicated by different symbols: metallic (gray circles), Mott-insulator (blue squares), and charge-disproportionated insulator (CDI, magenta triangles). The boundary of the CDI phase is fitted by the red line

We first establish the main overall effect of the interaction parameters *U* and *J* on the electronic properties of LuNiO_3_ within the high-symmetry *Pbnm* structure, i.e., $R_1^ + = 0.0\,{\text{{\AA}}}$. The resulting phase diagram is presented in Fig. 1. Analogously to ref. ^26^, we can identify three distinct phases: First, a standard Mott-insulating phase for large *U* values, with vanishing spectral weight around the Fermi level, *A*(*ω* = 0) = 0, and equal occupation of all Ni sites. Second, another insulating phase for moderate *U* values of around 2–3.5 eV and relatively large *J* $\left({>rsim 0.4\,{\mathrm{eV}}} \right)$, which is characterized by a strong difference in total occupation of the Wannier functions centered on LB and SB Ni sites, respectively (*n*_LB_ ≥ 1.5 and *n*_SB_ ≤ 0.5). We denote this phase as charge disproportionated insulating (CDI) phase.^35^ Third, a metallic phase for small *U* values in between the two insulating regions, with equal occupation on all Ni sites, *n*_*SB*_ ≈ *n*_*LB*_ ≈ 1.0, and nonvanishing spectral weight at the Fermi level, *A*(*ω* = 0) > 0.

The CDI phase has been identified as the insulating low-temperature phase of nickelates in ref. ^26^, where it has also been shown that the strong charge disproportionation is linked to the MIT (in ref. ^26^ this phase has been termed “bond disproportionated insulating”). We note that the Wannier basis within our low-energy subspace, while being centered on the Ni sites with strong *e*_*g*_ character, also exhibits strong tails on the O ligands, and thus the corresponding charge is distributed over the central Ni atom and the surrounding O atoms. The strong charge disproportionation found within our chosen basis set is thus fully consistent with the observation that the integrated charge around the two different Ni atoms differs only marginally.^23^ Alternatively, within a negative charge transfer picture, the MIT can also be described, using a more atomic-like basis, as $(d^8\underline L )_i(d^8\underline L )_j \to (d^8\underline L ^2)_{{\mathrm{SB}}}(d^8)_{{\mathrm{LB}}}$, where $\underline L$ denotes a ligand hole (c.f. Refs. ^23,29,36,37^).

One should also note that the CDI phase appears even though all Ni sites are structurally equivalent ($R_1^ + = 0$ in Fig. 1), which indicates an electronic instability toward spontaneous charge disproportionation. This has already been found in ref. ^26^, and indicates that a purely lattice-based description is incomplete. Moreover, within our CSC DFT + DMFT calculations, the CDI phase appears at significantly lower *J* and a more confined *U* range compared to the non-CSC calculations of ref. ^26^. A similar reduction of *J* values necessary to stabilize the CDI phase has also been achieved in the non-CSC DFT + DMFT calculations of ref. ^38^, through the introduction of an (effective) inter-site Hartree interaction. This suggests that the latter can indeed mimic the main effect of a CSC calculation, where the charge density, and thus the local occupations, are updated and the Hartree energy is recalculated in each CSC step.

**Fig. 2:**
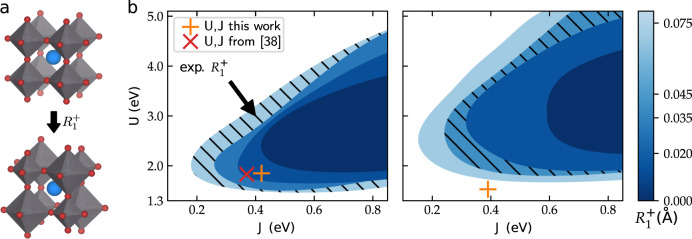
**a** Illustration of the $R_1^ +$ breathing mode distortion. **b** Extension of the CDI phase within the (*U*, *J*) phase diagram for varying $R_1^ +$ breathing mode amplitude for LuNiO_3_ (left) and PrNiO_3_ (right). Each $R_1^ +$ amplitude is represented by a different brightness level, according to the color scale on the right, starting from $R_1^ + = 0.0\,{\text{{\AA}}}$ (darkest) to $R_1^ + = 0.075\,{\text{{\AA}}}$ (brightest). The levels corresponding to the experimental $R_1^ +$ amplitudes for *R* = Lu^18^ and *R* = Pr,^66^ respectively, are highlighted by diagonal stripes. The obtained cRPA values for *U* and *J* are marked by orange crosses and compared to the values from ref. ^38^ for LuNiO_3_ (red diagonal cross)

Next, we investigate how the electronic instability corresponding to the CDI phase couples to the structural $R_1^ +$ breathing mode distortion. For this, we vary only the $R_1^ +$ amplitude, while keeping all other structural parameters fixed to the fully relaxed (within nonmagnetic DFT) *Pbnm* structures, and calculate (*U*, *J*) phase diagrams for different values of the $R_1^ +$ amplitude. We do this for both LuNiO_3_ and PrNiO_3_, i.e., for the two compounds with the smallest and largest rare earth cations within the series that exhibit the MIT. The (*U*, *J*) range of the CDI phase for a given $R_1^ +$ amplitude is then extracted by interpolating the convex hull of the phase boundary (similar to the red line in Fig. 1). The results are summarized in Fig. 2b.

In both cases, *R* = Lu and *R* = Pr, the $R_1^ +$ amplitude couples strongly to the CDI state, and increases the corresponding area within the (*U*, *J*) phase diagam. In particular, the minimal *J* required to stabilize the CDI phase is significantly lowered. Furthermore, also for *R* = Pr, there is a spontaneous instability toward the formation of a CDI state, but the corresponding (*U*, *J*) range is noticeably smaller than for *R* = Lu. In addition, the minimal *U* required to stabilize the CDI phase for a given $R_1^ +$ amplitude is slighty higher for *R* = Pr than for *R* = Lu. We note that, since the *R* ions do not contribute noticeably to any electronic states close to the Fermi level, the differences between the two materials are mainly due to the different underlying *Pbnm* structures, specifically the weaker octahedral tilts in PrNiO_3_ compared to LuNiO_3_. This increases the electronic bandwidth, which opposes the tendency toward charge disproportionation.

### Calculation of interaction parameters

So far we have varied *U* and *J* in order to obtain the general structure of the phase diagram. Next, we calculate *U* and *J* corresponding to our correlated subspace for all systems across the series to see where in these phase diagrams the real materials are located. We use cRPA^32^ to extract the partially screened interaction parameters (*U*, *J*) within the Hubbard–Kanamori parameterization, by separating off the screening channels related to electronic transitions within the correlated *e*_*g*_ subspace from all other transitions (see also Methods section).

**Fig. 3:**
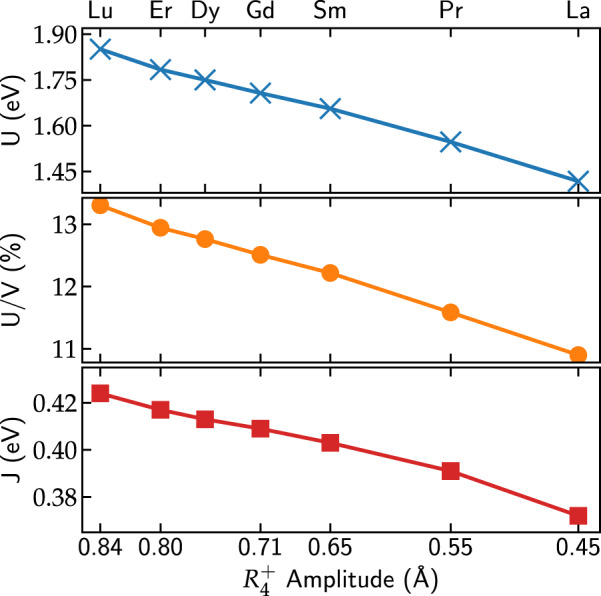
Screened onsite Hubbard–Kanamori interaction parameters *U* (top) and *J* (bottom) for the *e*_*g*_ orbitals within our low-energy subspace across the nickelate series as a function of the octahedral tilt amplitude $R_4^ +$. Additionally, the ratio between *U* and the corresponding bare (unscreened) interaction parameter *V* is shown (middle)

The results of these cRPA calculations are shown in Fig. 3 as a function of the *R* cation and the corresponding $R_4^ +$ amplitude, i.e., the main octahedral tilt mode in the *Pbnm* structure. The effective interaction parameters *U* corresponding to our *e*_*g*_ correlated subspace are strongly screened compared to the bare interaction parameters *V*. For LuNiO_3_, we obtain *V* = 13.91 eV and *U* = 1.85 eV, while *J* = 0.42 eV with a corresponding bare value of 0.65 eV. This is in good agreement with ref. ^38^, which obtained *U* = 1.83 eV and *J* = 0.37 eV using the experimental *P*2_1_/*n* structure. Furthermore, both *U* and *J* decrease monotonically across the series (for decreasing $R_4^ +$ amplitude), leading to an additional reduction of *U* by 25% in LaNiO_3_ compared to LuNiO_3_. This decrease is also observed in the ratio *U*/*V*, indicating that it is due to an even stronger screening for *R* = La compared to *R* = Lu.

Our calculated (*U*, *J*) parameters for *R* = Lu and *R* = Pr are also marked in the corresponding phase diagrams in Fig. 2. It is apparent, that for *R* = Lu the calculated cRPA values are well within the stability region of the CDI phase, even for a relatively small $R_1^ +$ amplitude of 0.02 Å. In contrast, for *R* = Pr, the values are outside the CDI phase even for $R_1^ +$ amplitudes larger than the one experimentally observed. Thus, at their respective experimental breathing mode amplitudes, our calculations predict a paramagnetic CDI state for LuNiO_3_ but not for PrNiO_3_.

### Lattice energetics

Up to now, we have been addressing the stability of the CDI phase for a given (fixed) $R_1^ +$ amplitude. Now, we will address the stability of the $R_1^ +$ mode itself and calculate its amplitude across the series using total energy calculations within CSC DFT + DMFT. The symmetry-based mode decomposition allows us to systematically vary only the $R_1^ +$ mode, while keeping all other structural parameters fixed to the values obtained from the nonmagnetic DFT calculations. Thus, in contrast to interpolation procedures as in refs. ^25,27^, our approach excludes any additional energy contributions related to simultaneous changes in other structural distortions, in particular the octahedral tilt modes.

**Fig. 4:**
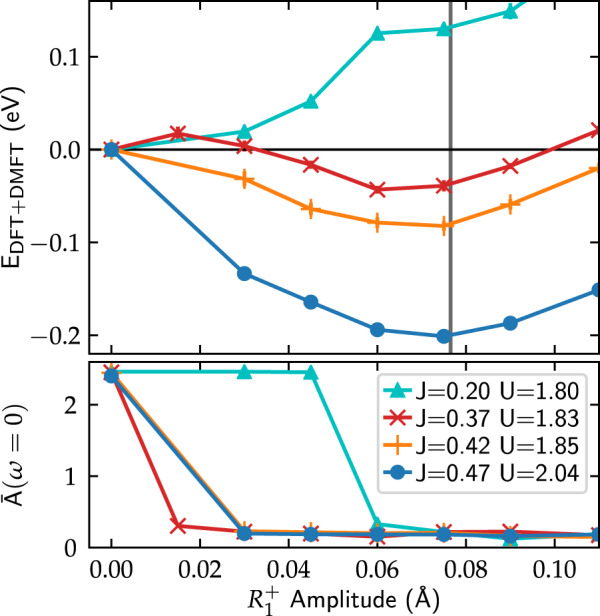
Top: Total energy, *E*_DFT+DMFT_, as a function of the $R_1^ +$ breathing mode amplitude for LuNiO_3_ using different values for the interaction parameters *U* and *J*. The experimental amplitude ($R_1^ + = 0.075\,{\text{{\AA}}}$^18^) is marked by the gray vertical line. Bottom: corresponding spectral weight at the Fermi level, indicating the MIT as a function of $R_1^ +$ amplitude

Figure 4 shows the total energy and the spectral weight around the Fermi level, $\bar A(\omega = 0)$, as a function of the $R_1^ +$ amplitude for LuNiO_3_, calculated using different values for (*U*, *J*). First, we focus on the results obtained using our cRPA calculated values (*J* = 0.42 eV, *U* = 1.85 eV, orange crosses). It can be seen, that the energy indeed exhibits a minimum for an $R_1^ +$ amplitude very close to the experimental value. Furthermore, as seen from $\bar A(\omega = 0)$, the system undergoes a MIT for increasing $R_1^ +$ amplitude and is clearly insulating in the region around the energy minimum. Thus, our CSC DFT + DMFT calculations together with the calculated cRPA interaction parameters correctly predict the CDI ground state for LuNiO_3_, and furthermore result in a breathing mode amplitude that is in excellent agreement with experimental data.

To see how subtle changes in (*U*, *J*) influence the energetics of the system, we also perform calculations using the cRPA values obtained in ref. ^38^ (*J* = 0.37 eV, *U* = 1.83 eV, red diagonal crosses). In this case, we obtain a more shallow energy minimum at a slightly reduced amplitude of $R_1^ + = 0.06\,{\text{{\AA}}}$. This reduction is mainly caused by the slightly smaller *J*. Moving the values of (*U*, *J*) even closer to the boundary of the stability region of the CDI phase for the experimental $R_1^ +$ amplitude, *cf*. Figure 2 (e.g., *J* = 0.2 eV, *U* = 1.8 eV, cyan triangles) results in a loss of the energy minimum for finite $R_1^ +$ amplitude. Nevertheless, a kink in the total energy is clearly visible at the $R_1^ +$ amplitude for which the system becomes insulating, indicating the strong coupling between the structural distortion and the MIT. A similar kink can also be recognized (for rather small $R_1^ +$ amplitude) in the total energy obtained for *J* = 0.37 eV and *U* = 1.83 eV, resulting in an additional local energy minimum at $R_1^ + = 0$, a typical hallmark of a first order structural transition. In addition, we also perform calculations where (*U*, *J*) are increased by 10% compared to our cRPA values (*J* = 0.47 eV, *U* = 2.04 eV, red circles), which leads to a deeper energy minimum and an $R_1^ +$ amplitude in near perfect agreement with experiment.

**Fig. 5:**
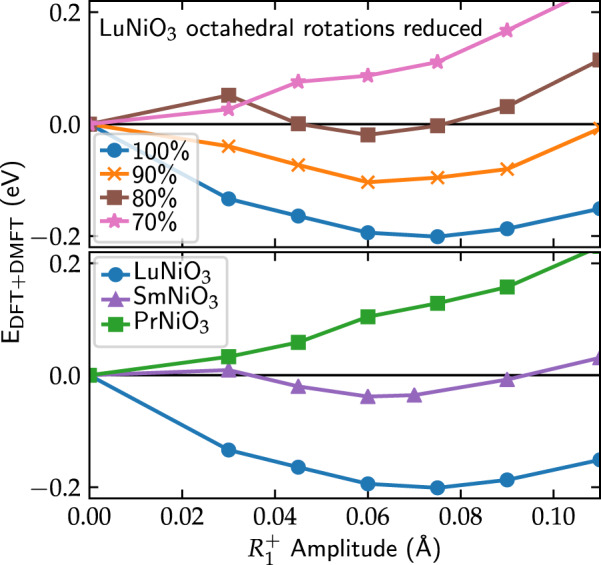
Top: Total energy as a function of the $R_1^ +$ breathing mode amplitude for LuNiO_3_ with octahedral rotation amplitudes reduced to 90, 80, and 70% (for *U* = 2.04 eV and *J* = 0.47 eV). Bottom: corresponding data for various materials across the nickelate series. Here, (*U*, *J*) values are increased by 10% compared to the results of the cRPA calculations (*U* = 2.04 eV/*J* = 0.47 eV for LuNiO_3_, *U* = 1.82 eV/*J* = 0.44 eV for SmNiO_3_, and *U* = 1.70 eV/*J* = 0.43 eV for PrNiO_3_)

Next, we investigate the influence of the octahedral rotations on the energetics of the $R_1^ +$ mode, where we perform a series of calculations for LuNiO_3_ with artificially decreased octahedral rotations (see Methods section), fixed (*U*, *J*), and fixed volume. As can be seen from the data shown in the top panel of Fig. 5, decreasing the amplitude of the octahedral rotations to 70%, which corresponds roughly to the amplitudes found for PrNiO_3_, leads to a vanishing of the minimum at nonzero $R_1^ +$ amplitude. This confirms that the reduction of the octahedral rotation amplitudes plays a crucial role in the energetics of the breathing mode distortion and in determining the trend across the nickelate series.

Finally, we examine how the energetics of the $R_1^ +$ mode varies across the series, by comparing the two end members LuNiO_3_ and PrNiO_3_, as well as SmNiO_3_, which is the compound with the largest *R* cation in the series that still exhibits a paramagnetic CDI state. In each case we use (*U*, *J*) values that are increased by 10% relative to the corresponding cRPA values. The use of such slightly increased interaction parameters is motivated by the observation that the *U* values obtained from the static limit of the (frequency-dependent) screened cRPA interaction are often too small to reproduce experimental data for various materials.^31,39–41^ The results are depicted in Fig. 5.

As discussed above, for LuNiO_3_ (blue circles), we obtain an energy minimum exactly at the experimentally observed amplitude. For SmNiO_3_ (purple triangles), we obtain a much more shallow minimum at $R_1^ + = 0.06\,{\text{{\AA}}}$, which corresponds to a reduction by ≈20% compared to LuNiO_3_. Unfortunately, structural refinements for SmNiO_3_ are only available within the *Pbnm* space group, and thus no information on the $R_1^ +$ amplitude exists.^42^ However, the reduction of the $R_1^ +$ amplitude from *R* = Lu to *R* = Sm is much more pronounced compared to previous DFT + *U* calculations with AFM order,^30^ where the reduction is only about 8%.

For PrNiO_3_ (green squares), no stable $R_1^ +$ amplitude is obtained within our paramagnetic DFT + DMFT calculations, but a kink marking the MIT is still visible at $R_1^ + = 0.06\,{\text{{\AA}}}$. This is also in agreement with the experimental observation that no paramagnetic CDI phase occurs in PrNiO_3_.^9^ Furthermore, it was recently demonstrated using DFT + DMFT calculations that for NdNiO_3_ the CDI state becomes only favorable in the antiferromagnetically ordered state.^27^ Our results indicate that this also holds for PrNiO_3_, while in SmNiO_3_ a stable $R_1^ +$ amplitude can be found even in the paramagnetic case. Thus, the phase boundaries across the series are correctly described within the DFT + DMFT approach. We further note that, considering the (*U*, *J*) phase diagrams for PrNiO_3_ in Fig. 2, a *U* of up to 2.5 or even 3 eV would be required to put PrNiO_3_ well within the CDI phase region at its experimental $R_1^ +$ amplitude, which appears necessary to obtain a stable $R_1^ +$ amplitude. However, such a large *U* seems highly unrealistic considering the calculated cRPA values.

## Discussion

In summary, the successful application of CSC DFT + DMFT and symmetry-based mode analysis, without ad hoc assumptions regarding the strength of the Hubbard interaction or fixing structural parameters to experimental data, allows to elucidate the nature of the coupled electronic–structural transition across the nickelate series. Our analysis reveals that the MIT, which is related to an electronic instability towards spontaneous charge disproportionation, leads to a significant restructuring of the energy landscape, indicated by a kink in the calculated total energy. This creates a minimum at a finite $R_1^ +$ amplitude (for appropriate *U* and *J*), and suggests a first order character of the coupled structural and electronic transition in the PM case, in agreement with experimental observations^11^ for both SmNiO_3_^43^ and YNiO_3_.^15^ We note that, since a certain critical value of $R_1^ +$ is necessary to induce the MIT (see, e.g., Fig. 4), a second order structural transition would imply the existence of an intermediate structurally distorted metallic phase, inconsistent with experimental observations.

The strength of the electronic instability towards spontaneous charge disproportionation and thus the stability range of the CDI phase, is strongly affected by the amplitude of the octahedral rotations, varying across the series. This is in agreement with ref. ^28^, but in addition we show that to arrive at a fully coherent picture, with correct phase boundaries, it is crucial to treat both electronic and structural degrees of freedom on equal footing. For example, even though a CDI state can be obtained for PrNiO_3_ for fixed $R_1^ +$ amplitude >0.06 Å, our calculations show that this is indeed energetically unstable. In addition, the octahedral rotations also influence the screening of the effective interaction parameters, disfavoring the CDI state for larger *R* cations. As a result, magnetic order appears to be crucial to stabilize the breathing mode distortion for both *R* = Nd and Pr.

Moreover, our calculations not only lead to a coherent picture of the MIT, but also allow to obtain accurate structural parameters across the nickelate series. Furthermore, this is achieved using only a minimal correlated subspace. We note that the use of such a reduced correlated subspace can be advantageous, since it not only allows to reduce the computational effort (due to less degrees of freedom), but also because the double-counting problem is typically less severe if the O-*p* dominated bands are not included in the energy window of the correlated subspace.^44,45^ In the present case, the resulting more extended Wannier functions, which also incorporate the hybridization with the surrounding ligands, also provide a rather intuitive picture of the underlying charge disproportionation.

Finally, our study represents the successful application of a combination of several state-of-the-art methods that allows to tackle other open issues related to the entanglement of structural and electronic properties in correlated materials, such as Jahn–Teller and Peierls instabilities, charge density wave, or polarons.

## Methods

### DFT calculations

All DFT calculations are performed using the projector augmented wave (PAW) method^46^ implemented in the “Vienna Ab initio Simulation Package” (VASP)^47–49^ and the exchange correlation functional according to Perdew, Burke, and Ernzerhof.^50^ For Ni, the 3*p* semicore states are included as valence electrons in the PAW potential. For the rare-earth atoms, we use PAW potentials corresponding to a 3+ valence state with *f*-electrons frozen into the core and, depending on the rare-earth cation, the corresponding 5*p* and 5*s* states are also included as valence electrons. A *k*-point mesh with 10 × 10 × 8 grid points along the three reciprocal lattice directions is used and a plane wave energy cut-off of 550 eV is chosen for the 20 atom *Pbnm* unit cell. All structures are fully relaxed, both internal parameters and lattice parameters, until the forces acting on all atoms are smaller than 10^−^^4^ eV/Å. As in ref. ^30^, we perform calculations for LaNiO_3_ within the *Pbnm* and *P*2_1_/*n* space groups, to allow for a more consistent comparison with the rest of the series, even though LaNiO_3_ is experimentally found in a different space group (*R*3̄*c*). See also the discussion in ref. ^22^.

### Distortion mode analysis

For the symmetry-based mode decomposition^33^ we use the software ISODISTORT.^51^ Thereby, the atomic positions within a distorted low-symmetry crystal structure, $\vec r_i^{{\mathrm{dist}}}$, are written in terms of the positions in a corresponding non-distorted high-symmetry reference structure, $\vec r_i^0$, plus a certain number of independent distortion modes, described by orthonormal displacement vectors, $\vec d_{im}$, and corresponding amplitudes, *A*_*m*_:

1 $$\vec r_i^{{\mathrm{dist}}} = \vec r_i^0 + \mathop {\sum}\limits_m A_m\vec d_{im}.$$

The distortion modes of main interest here are the out-of-phase and in-phase tilts of the oxygen octahedra, $R_4^ +$ and $M_3^ +$, for characterization of the high-temperature *Pbnm* structure, and the $R_1^ +$ breathing mode distortion within the low-temperature *P*2_1_/*n* structure. A more detailed description for nickelates can be found, e.g., in refs. ^19,30^. For the calculations with reduced octahedral rotation amplitudes shown in Fig. 5, both $R_4^ +$ and $M_3^ +$ modes, as well as the $X_5^ +$ mode intimately coupled to these two modes, have been reduced by a common factor.

### DMFT calculations

The Wannier functions for our CSC DFT + DMFT calculations are constructed via projections on local Ni *e*_*g*_ orbitals as described in ref. ^52,53^, using the TRIQS/DFTTools software package.^54,55^ The effective impurity problems within the DMFT loop are solved with the TRIQS/cthyb continuous-time hybridization-expansion solver,^56^ including all off-diagonal spin-flip and pair-hopping terms of the interacting Hubbard–Kanamori Hamiltonian.^57^ The LB and SB Ni sites are treated as two separate impurity problems (even for zero $R_1^ +$ amplitude), where the number of electrons per two Ni sites is fixed to 2, but the occupation of each individual Ni site can vary during the calculation (while the solution is constrained to remain paramagnetic).

The fully localized limit^58^ is used to correct for the double-counting (DC) in the parametrization given in ref. ^59^:

2 $$\Sigma _{dc,\alpha }^{imp} = \bar U\left( {n_\alpha - \frac{1}{2}} \right),$$

where *n*_*α*_ is the occupation of Ni site *α*, obtained in the DMFT loop, and the averaged Coulomb interaction is defined as $\bar U = (3U - 5J)/3$. Note, that in our Wannier basis the occupations change quite drastically from the original DFT occupations and the choice of the DC flavor can therefore influence the outcome. However, with respect to the lattice energetics we found no difference in the physics of the system when changing the DC scheme or using fixed DFT occupation numbers for the calculation of the DC correction. If the DFT occupations are used instead of the DMFT occupations, larger interaction parameters are required to obtain the same predicted $R_1^ +$ amplitude. However, we note that the DFT occupations have no clear physical meaning within CSC DFT + DMFT.

The spectral weight around the Fermi level, $\bar A(\omega = 0)$, is obtained from the imaginary time Green’s function^60^:

3 $$\bar A(\omega = 0) = - \frac{\beta }{\pi }G_{{\mathrm{imp}}}\left( {\frac{\beta }{2}} \right).$$

For *T* = 0 (*β* → ∞), $\bar A$ is identical to the spectral function at *ω* = 0. For finite temperatures, it represents a weighted average around *ω* = 0 with a width of ~*k*_B_*T*^60^.

The total energy is calculated as described in ref. ^31^:

4 $$\begin{array}{rcl} E_{{\mathrm{DFT}} + {\mathrm{DMFT}}} & =& E_{{\mathrm{DFT}}}[\rho ] \\ &&  - \frac{1}{N_{k}} \mathop{\sum}\limits_{\lambda, {\vec{k}}} \varepsilon_{\lambda, {\vec{k}}}^{{\mathrm{KS}}}\,f_{\lambda {\vec{k}}} + \langle H_{{\mathrm{KS}}}\rangle _{{\mathrm{DMFT}}} \\ &&  + \langle H_{{\mathrm{int}}}\rangle _{{\mathrm{DMFT}}} - E_{{\mathrm{DC}}}^{imp}. \end{array}$$

The first term is the DFT total energy, the second term subtracts the band energy of the Ni-*e*_*g*_ dominated bands (index *λ*), the third term evaluates the kinetic energy within the correlated subspace via the lattice Green’s function, the fourth term adds the interaction energy, where we use the Galitskii–Migdal formula,^61,62^ and the last term subtracts the DC energy. To ensure good accuracy of the total energy, we represent both *G*_imp_ and Σ_imp_ in the Legendre basis^63^ and obtain thus smooth high-frequency tails and consistent Hartree shifts. Moreover, we sample the total energy over a minimum of additional 60 converged DMFT iterations after the CSC DFT + DMFT loop is converged. Convergence is reached when the standard error of the Ni site occupation of the last 10 DFT + DMFT loops is smaller than 1.5 × 10^−3^. That way we achieve an accuracy in the total energy of <5 meV. All DMFT calculation are performed for *β* = 40 eV^−1^, which corresponds to a temperature of 290 K.

### cRPA calculations

We use the cRPA method as implemented in the VASP code^64^ to extract interaction parameters for our correlated subspace. These calculations are done for the relaxed *Pbnm* structures.^30^ We follow the ideas given in the paper of ref. ^26^ and construct maximally localized Wannier functions (MLWFs) for the Ni-*e*_*g*_ dominated bands around the Fermi level using the wannier90 package.^65^ Since the corresponding bands are isolated from other bands at higher and lower energies, no disentanglement procedure is needed, except for LaNiO_3_, for which we ensured that the resulting Wannier functions are well converged and have a very similar spread as for all other compounds of the series.

We divide the total polarization, *P*, into a contribution involving only transitions within the effective “*e*_*g*_” correlated subspace and the rest, $P = P_{e_g} + P_r$. The constrained polarization, *P*_*r*_, and the static limit of the screened interaction matrix, *W*_*r*_(*ω* = 0) = *V*[1−*VP*_*r*_(*ω* = 0)]^−1^, where *V* is the bare interaction, are then calculated using a 5 × 5 × 3 *k*-point mesh, a plane wave energy cut-off of *E*_cut_ = 600 eV, and 576 bands. Effective values for the Hubbard–Kanamori interaction parameters (*U*, *J*) are extracted from *W*_*r*_(*ω* = 0) as described in ref. ^57^. Our procedure is analogous to the calculation of effective interaction parameters for LuNiO_3_ in ref. ^38^.

It should be noted that the MLWFs used for the cRPA calculations are not completely identical to the projected Wannier functions used as basis for the correlated subspace within our DMFT calculations. However, test calculations for the case of LuNiO_3_ showed only minor differences between the hopping parameters corresponding to the MLWFs and the ones corresponding to the Wannier functions generated by the projection scheme implemented in VASP. Furthermore, we did not find a noticeable difference between the screened (*U*, *J*) values calculated for the MLWFs and the ones calculated for the initial guesses for these Wannier functions, i.e., before the spread minimization, which are also defined from orthogonalized projections on atomic-like orbitals. We thus conclude that the two sets of Wannier functions are indeed very similar, and that the cRPA values of (*U*, *J*) obtained for the MLWFs are also representative for the Wannier basis used in our DMFT calculations.

Additionally, we point out that, in contrast to what was found in ref. ^38^, we observe only negligible differences in the interaction parameters obtained for the relaxed *Pbnm* structure and the ones obtained for the experimental low-temperature *P*2_1_/*n* structure for LuNiO_3_ (1.827 eV and 1.876 eV compared to 1.849 eV within *Pbnm*). In particular, the difference of the interaction parameters on the two inequivalent Ni sites in the *P*2_1_/*n* structure (±0.03 eV) are very small compared to the changes stemming from different degrees of octahedral rotations (i.e., different *R* cations), justifying the use of constant interaction parameters for different $R_1^ +$ amplitudes. Furthermore, the differences in the intra-orbital *U* matrix elements between the $d_{z^2}$ and the $d_{x^2 - y^2}$ orbitals are negligible small, ~0.01 eV, in our calculations. Therefore, all the values of the interaction parameters are averaged over both *e*_*g*_ orbitals.

## Data Availability

The data that support the findings of this study are available from the corresponding author upon reasonable request.
